# Substance basis and pharmacological mechanism of heat-clearing herbs in the treatment of ischaemic encephalopathy: a systematic review and network pharmacology

**DOI:** 10.1080/07853890.2024.2308077

**Published:** 2024-01-29

**Authors:** Andong Zhao, Qianqian Sun, Jiahao Zhang, Tian Hu, Xuewei Zhou, Chuan Wang, Jiping Liu, Bin Wang

**Affiliations:** a Pharmacology of Chinese Medicine, Shaanxi University of Chinese Medicine, Xianyang, China; b Key Laboratory of Pharmacodynamics and Material Basis of Chinese Medicine of Shaanxi Administration of Traditional Chinese Medicine, Xianyang, China; c Engineering Research Center of Brain Health Industry of Chinese Medicine, Universities of Shaanxi Province, Xianyang, China

**Keywords:** Ischaemic encephalopathy, heat-clearing Chinese medicines, network pharmacology, active ingredients, signalling pathway

## Abstract

**Background and objective:**

Ischaemic encephalopathy is a common cerebrovascular disease caused by insufficient blood supply to the cerebral vessels. The ischaemic encephalopathy is closely associated with the development of many chronic diseases such as obesity, hypertension and diabetes. Neurotrophic therapy has become the main therapeutic strategy for ischaemic encephalopathy. However, neurotrophic drugs only slightly recover the neurological function of patients, and their long-term efficacy is uncertain. Previous reports revealed that the active ingredients of natural medicines play important roles in the treatment of cerebral ischemia. In this study, we reviewed clearing herbs with anti-ischaemic encephalopathy functions using the data from quantitative statistical and network pharmacological exploration methods. We also discussed the different bioactive components and pharmacological effects of these herbs.

**Methods:**

First, we collected Chinese herbal prescriptions against ischaemic encephalopathy in four databases. Then, we statistically analysed the frequency of application of heat-clearing herbs to obtain the commonly used heat-clearing herbs against ischaemic encephalopathy, and classified them according to their efficacy according to the statistical results, to summarize the mechanism of anti-ischaemic effects of different bioactive components; Second, the network database was used to obtain the above components of heat-clearing Chinese medicines and their corresponding targets of action, disease targets of ischaemic stroke; Venny 2.1.0 was used to obtain component-disease target intersections; Cytoscape was used to construct the ‘Drug-Active Ingredient-Target Network Graph ‘; DAVID was used for GO and KEGG enrichment analysis.

**Results:**

Literature and database screening involved 149 prescriptions, with a total of 269 flavours of Chinese medicines and 20 flavours of single-flavour heat-clearing Chinese medicines; The top nine in terms of frequency of use were Radix Paeoniae Rubra、Rehmanniae Radix Praeparata、Figwort Root、Cortex Moutan、Scutellariae Radix、Coptidis Rhizoma、Gardeniae Fructus、Cassiae Semen、Lonicerae Japonicae Flos. The common components obtained from network pharmacology were beta-sitosterol, quercetin, and stigmasterol, which mainly act on key targets such as RELA, AKT1, JUN, PRKACA, PTGS2, RAF1 and CHUK; and their active ingredients are mainly involved in signalling pathways such as Calcium, PI3K-Ak, MAPK, cAMP, IL-17, HIF-1, TNF, T-cell receptor, NF-kappa B and JAK-STAT.

**Conclusions:**

Heat-clearing herbs are useful and promising for the protection against and prevention of ischemic encephalopathy. The results of the network pharmacological studies are similar to the mechanisms of anti-ischemic encephalopathy of the active ingredients of the purgative herbs we have listed; Thin either directly protects cerebrovascular tissues by improving vascular permeability and reducing the area of infarcted tissues, or produces protective effects through molecular signaling pathways. It can be seen that the components of heat-clearing Chinese medicines can exert cerebroprotective effects through multiple pathways, which provides us with a reference for further development and study of heat-clearing Chinese medicines in the treatment of ischemic cerebrovascular diseases.

## 1. Introduction

Ischaemic cerebrovascular illness is the cause of temporary or permanent cerebrovascular insufficiency of the blood supply to a particular brain region, including acute ischaemic stroke; transient ischaemic attack, chronic cerebral circulatory insufficiency, acute cerebral venous sinus thrombosis and chronic cerebrospinal venous insufficiency [1]. Ischaemic encephalopathy is characterized by high levels of disability, fatalities and recurrence. Its post-onset mortality rate is the second highest among all diseases. Thus, ischemic encephalopathy poses putting a significant threat to human life. In addition, ischaemic encephalopathy negatively affects the quality of life and places a huge economic burden on the family of patients and society. According to the Global Burden of Disease study, 2.87 million new cases of ischaemic stroke were reported in China in 2019 [2], representing approximately 73% of the total number of stroke cases, of which 1.03 million, or approximately 36%, died from ischemic cerebrovascular disease. This report also showed that from 1990 to 2019, the age-specific incidence of stroke decreased by 9.3%, mainly due to cerebral and subarachnoid haemorrhages, which decreased by 53.1% and 39.3%, respectively. In contrast, the age-specific incidence of ischaemia stroke increased by 34.7%. Numerous epidemiological studies have reported a strong association between ischaemic cerebrovascular disease and diabetes, hypertension, obesity and kidney disease, suggesting that ischaemic cerebrovascular disease is likely to increase in the upcoming years. Therefore, developing new drugs for treating ischaemic cerebrovascular diseases is urgently necessary.

The pathogenesis of ischaemic encephalopathy is complex and mainly related to local ischaemia associated with clots within the cerebral arteries [2,3]. Cerebral thrombosis is often originated from lesions in the arterial vascular wall, usually observed as atherosclerosis and plaques. Cerebral thrombosis causes various pathological responses, including excitatory amino acid toxicity in the brain tissue, mitochondrial dysfunction, oxidative stress, apoptosis and inflammatory responses. Immediately after cerebral ischaemia, the decreased hematopoietic flow, lack of sugars, oxygen and adenosine triphosphate (ATP), induce depolarization and glutamate release in neuronal cells. Then, glutamate stimulates the Na^+^/Ca^2+^ channels coupled to N-methyl-D-aspartate receptors (NMDARs), Enhanced Ca^2+^ inward flow disrupts ion homoeostasis, leading to Ca^2+^ overload in mitochondria and cytoplasm. NMDARs containing GluN2B subunits are also activated and variations in these stimulate several peptidases, lipases, aliases, phosphatases and other enzymes, which triggers excitotoxicity. In addition, Ca^2+^ influx and increased reactive oxygen species (ROS) production following hypoxia, open the mitochondrial permeability transition pore (MPTP) and release cytochrome c, resulting in mitochondrial dysfunction. Ca^2+^ overload also ­activates calpain, disrupting the fission of B-cell lymphoma-2 (Bcl-2), and allowing BH3-interacting domain death agonis (BID) to react with caspase-8 within the death acceptor pathway, transforming it into a triggered active form. The interaction of truncated BID with members of the anti-apoptotic Bcl-2 protein family results in dimerization and initiation of MPTP, leading to the release of different pro-apoptotic agents, including cytochrome c, endonuclease G and apoptosis-inducing factor (AIF), and the establishment of apoptotic bodies through conjugation with the activators of apoptotic proteases.

After the apoptosome formation, caspase-9 is activated, triggering lower downstream caspases, including caspase-3, caspase-6 and caspase-7, which promotes the apoptosis in neuronal cells, which is part of the intrinsic (or mitochondrial) route of apoptosis, and the exogenous (or death receptor) way, a initiated by the binding of certain ligands, such as tumour necrosis factor (TNF)-α, Fas ligand (FasL) and TNF-related apoptosis-inducing ligand (TRAIL) to the respective death acceptors on the cell wall (TNF-α, Fas/CD95/APO1 and TRAIL-r, respectively). During ischaemia, the receptors recruit the proteins Fas-associated death domain (FADD) and TNF receptor type 1-associated death domain (TRADD), inducing various downstream damage processes by binding to procaspases to form complexes that ultimately lead to caspase8 activation, caspase-8 triggering downstream effector caspases and finally apoptosis.

**Figure 1.**
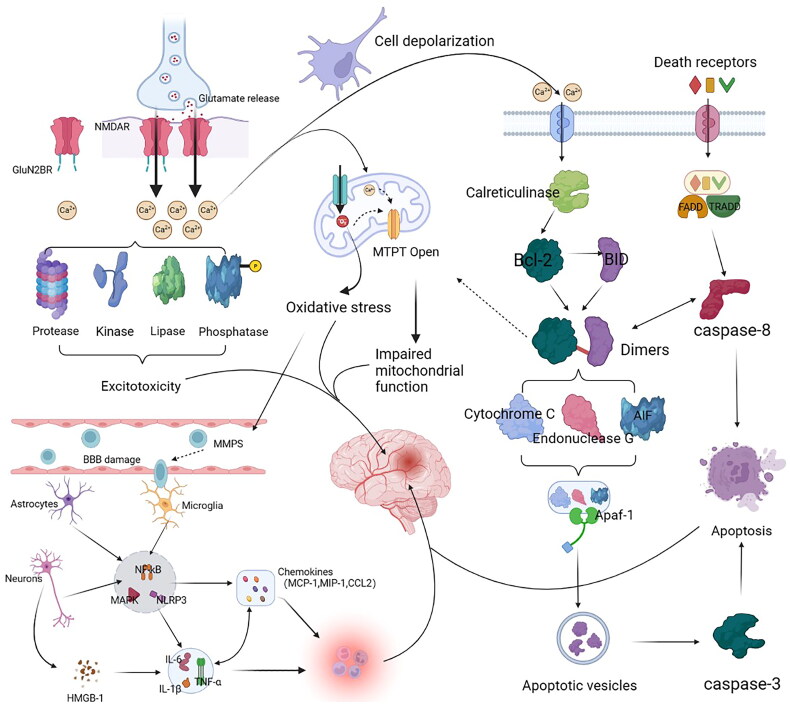
Diagram of the pathogenesis of cerebral ischaemia.

Microglia are the primary permanent immune cells in the brain and are the first ones recruited to the infarct site. Microglia secretes hyperinflammatory cell factors like interleukin (IL)-1β, IL-6 and TNF-α that immediately stimulate the production of chemokines, such as monocyte chemoattractant protein-1 (MCP-1), follalkine, giant phagocyte infectious protein 1, microglial reactive factor-1 and neutrophil chemotactic agents induced by cytokines. Enhanced chemokine expression exacerbates ischemic injury. High mobility group box protein 1 (HMGB1) concentrations during neuroinflammation are dramatically increased in the brain, particularly in glia, adipocytes, and vascular stores, where these cells are strongly linked to inflammation and acellular stress. Pericellular HMGB1 and toll-like receptor (TLR)2 or TLR4 interact and then interacts with factor nuclear kappa B (NF-κB) to trigger immune responses. In addition, HMGB1 promotes the production of several pro-inflammatory cytokines, among them inducible nitric oxide synthase (NOS), cytochrome c, oxidase subunit 2, IL-1β, and TNF-α, triggering cell death of neuronal cells in ischaemia. The mitogen-activated protein kinase (MAPK) pathway also induces the production of pro-inflammatory factors. MAPK includes three major effectors: extracellular signal-regulated kinase (ERK)1/2, c-Jun N-terminal kinase (JNK) and p38 stress-activated factor kinase, which play deleterious roles in cerebral ischemia. The stimulation of matrix metalloproteinase (MMP) expression by activating MAPK/ERK signalling exacerbates blood-brain barrier injury in ischemic stroke and enhances the production of pro-inflammatory elements (Figure 1).

Chinese medicine has significant advantages in preventing and treating ischemic cerebrovascular diseases, with remarkable results in prevention, treatment, health care and rehabilitation. Chinese medicines exert therapeutic effects on several biological activities and pathways, including energy metabolism, oxidative stress, inflammation, and apoptosis and have significant advantages over traditional single compounds. Moreover, multi-targeted drugs avoid the over-regulation of one target or signalling pathway and theoretically have fewer side effects. Professor Wang Yongyan, a modern medical doctor, proposed the theory of ‘poison damages the brain ligaments’ [4], which suggests that mucus and stagnant blood accumulate in the brain, creating internal poison and damaging the brain ligaments. Yongyan highlighted that heat and toxicity are common in the acute phase of stroke and that preventing heat and toxicity from damaging the brain and ligaments is key to controlling the disease progression. Therefore, clearing heat and detoxifying toxins are the gold standard for treating of toxin-damaged cerebral ligaments.

Heat-clearing herbs are traditionally classified into five categories based on their efficacy: heat-clearing and detoxification, heat-clearing and cooling of the blood; heat-clearing and fire-removing; heat-clearing and dampness-drying; and heat-clearing for deficiency. Purgatives ameliorate ischemic strokes. Pufa [5] showed that clearing heat, cooling blood, and clearing blood stasis improve haemodynamics and relieve the clinical symptoms of patients in the emergency stage of ischemic stroke, with satisfactory efficacy and safety. Tao et al. [6] demonstrated that clearing heat, dissolving mucus, and removing toxins reduced cerebral oedema and enhanced cerebral nerve function in rats with ischemic stroke. This method of clearing heat and detoxifying toxins has and ideal application value for treating ischaemic brain vascular disease. Heat-clearing herbs are used to remove the heat and poison using their cooling properties to interrupt internal heat and poison pathogenesis. This is valuable in preventing and treating ischemic cerebrovascular disease.

## 2. Materials and methods

### 2.1. Analysis of the pattern of drug use of heat-clearing herbs for ischaemic stroke

Recently, with the extraction of Chinese medicine ingredients to provide strong evidence of the development of Chinese medicine, and to provide a reference for Chinese medicine clinical personnel to study or apply Chinese medicine in the treatment of ischaemic cerebrovascular diseases, this paper adopts the statistical method of information quantification to conduct statistical analysis for the frequency of using Chinese medicine in ischemic cerebrovascular diseases. According to the statistical results, the study was classified and analysed according to the efficacy of Chinese medicine in the category of clearing heat to better guide the results were classified according to the efficacy of heat-clearing herbs to better guide clinical use.

### 2.2. Search strategy

**Figure 2.**
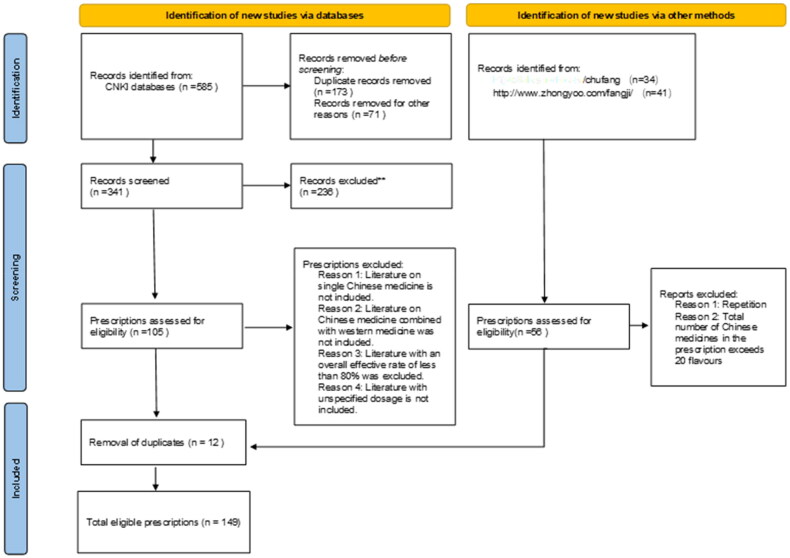
Prescription screening flow chart.

In the CNKI database, ‘Ischaemic stroke,’ ‘Cerebral ischemia’ and ‘Cerebral infarction’ were selected as the subject words, and ‘Treatment of traditional Chinese medicine’ was selected as the sub-subject words. The years of literature search were from 1994 to 2023, and a total of 585 articles were searched; Using the ‘Prescription Database for Proprietary Chinese Medicines’ (https://db.yaozh.com/chufang) and the Complete Formulary (http://www.zhongyoo.com/fangji/) as search tools, we searched for ‘ischaemic stroke’, ‘cerebral ischaemia’ and ‘cerebral infarction’, and 49 prescriptions were obtained; Inclusion criteria: literature on the clinical treatment of cerebral ischemia with Chinese herbal compound was selected; literature on cerebral ischemia caused by other chronic diseases such as diabetes was excluded; literature on single herbal medicine was excluded; literature on the treatment of Chinese herbal medicine in combination with western medicine was excluded; literature on the overall efficiency of treatment was excluded if it was less than 80%; literature on the absence of a clear dose of medicine was excluded. A total of 149 formulae were collated and recorded as 149 data records. The literature search and screening processes are shown in Figure 2.

### 2.3. Analysis of literature

There are many different types of active ingredients in Chinese medicines and their pharmacological mechanisms of action are complex. Volatile oils, alkaloids, organic acids, flavonoids, polysaccharides, and terpenoids are the main components used to treat ischemic stroke. The pharmacological mechanism is mainly through anti-inflammatory, anti-oxidant, anti-apoptotic and anti-oedema effects. Through the elaboration of the pathological mechanism of ischaemic stroke and the elucidation of the basic theories of Chinese medicine, it provides new ideas to reveal the material basis and mechanism of action of TCM in the treatment of ischemic stroke.

### 2.4. Network pharmacological analysis

#### 2.4.1. Data sources

**Table:** 

databases	Websites
Traditional Chinese Medicine Systems Pharmacology Database and Analysis Platform	https://old.tcmsp-e.com/tcmsp.php
The Human Gene Database	https://www.genecards.org/
The Comparative Toxicogenomics Database	https://ctdbase.org/
Therapeutic Target Database	https://db.idrblab.net/ttd/
UniProt	http://www.uniprot.org/
Venny2.1.0	https://bioinfogp.cnb.csic.es/tools/venny/index.html
DAVID Bioinformatics Resources	http://david.abcc.ncifcrf.gov
STRING	https://www.string-db.org/
Cytoscape-3.7.2	https://cytoscape.org/

#### 2.4.2 Collection and screening of components and targets

The TCMSP database was searched for all the components of Radix Paeoniae Rubra、Rehmanniae Radix Praeparata、Figwort Root、Cortex Moutan、Scutellariae Radix、Coptidis Rhizoma、Gardeniae Fructus、Cassiae Semen、Lonicerae Japonicae Flos respectively with the search terms, and the results were screened by OB ≥ 30% and DL ≥ 0.18, and the resulting components were imported into Excel sheets to establish a library of easily absorbed compounds of heat-clearing traditional Chinese medicines.

The potential targets related to each active Ingredient were collected in the TCMSP platform, and the obtained protein target information was input into the UniProt database, selecting the species as ‘human,’ removing duplicates and unvalidated targets, and importing the screening results into Excel to establish a target information database for the active ingredient genes.

Using ‘cerebral ischaemic stroke’ as the search term, we collected ischemic encephalopathy-related targets from the CTD, TTD, and GeneCards databases, merged the results of the three databases, and established a database of ischemic encephalopathy-related targets by removing duplicate targets.

#### 2.4.3. Network diagram construction and GO enrichment analysis

Venny2.1 was used to intersect the relevant targets of each heat-clearing herbal medicine with the relevant targets of CIS and to establish a database of gene targets of heat-clearing herbal medicines against ischemic encephalopathy.

The anti-ischemic encephalopathy targets of heat-clearing traditional Chinese medicines were uploaded to the DAVID platform for GO biological function annotation and KEGG pathway enrichment analysis, and the top 10 GO biological functions and top 20 KEGG pathways were screened.

#### 2.4.4. Construction of drug component-target-pathway interaction network diagrams

The screened active ingredients, target genes, and pathways were organized into network and type table files according to their relevance, and imported into Cytoscape 3.7.2 software to construct the drug ingredient-target-pathway interaction diagram. Using the NtheetoworkAnalyzer plug-in, a network degree analysis was performed to analyse the efficacy of heat-clearing traditional Chinese medicines in the treatment of CIS.

## 3. Results

### 3.1. Quantitative information statistics

#### 3.1.1. Screening results

A total of 149 prescriptions were included after screening. Statistical analysis revealed, 269 Chinese herbal medicines in this database, including 20 single herbal medicines for clearing heat. The top 10 in terms of frequency of use were Radix Paeoniae Rubra、Rehmanniae Radix Praeparata、Figwort Root、Cortex Moutan、Scutellariae Radix、Coptidis Rhizoma、Gardeniae Fructus、Cassiae Semen、Lonicerae Japonicae Flos and Cornu Bubali, the specific frequency of use is shown in Table 1.

**Table 1.** Single herbs for clearing heat in the treatment of ischemic stroke.

No.	Traditional Chinese Medicine	Frequency	Drug Class	four natures and five flavors of drugs	meridian entry
1	Gardeniae Fructus	5	clear away heat and purge pathogenic fire	bitter、cold	heart、lung、triple burner
2	Cassiae Semen	4		sweet、bitter、salty、cold	liver、large intestine
3	Gypsum	2		sweet、pungent、cold	lung、stomach
4	Prunellae Spica	2		bitter、pungent、cold	liver、gallbladder
5	Calcitum	1		pungent、salty、cold	lung、stomach、kidney
6	Scutellariae Radix	13	clearing heat and drying damp	bitter、cold	lung、gallbladder、spleen、large intestine
7	Coptidis Rhizoma	12		bitter、cold	heart、stomach、liver、gallbladder、spleen、large intestine
8	Gentiana cruciata	1		bitter、cold	liver、gallbladder
9	Lonicerae Japonicae Flos	5	clearing away heat and toxic material	sweet、cold	lung、heart、stomach
10	Forsythiae Fructus	2		bitter、cold	lung、heart、small intestine
11	Violsse Herba	1		bitter、pungent、cold	heart、liver
12	Fel Ursi	1		bitter、cold	liver、gallbladder、heart
13	Rhapontici Radix	1		bitter、salty、cold	stomach、large intestine
14	Radix Paeoniae Rubra	41	removing pathogenic heat from blood	bitter、cold	liver、spleen
15	Rehmanniae Radix Praeparata	10		sweet、cold	heart、liver、kidney
16	Cornu Bubali	7		bitter、cold	heart、liver
17	Figwort Root	5		sweet、bitter、salty、cold	lung、stomach、kidney
18	Cortex Moutan	5		bitter、pungent、cold	heart、liver、kidney
19	*Artemisia annua* L.	1	clearing deficient heat	bitter、pungent、cold	liver、gallbladder、kidney
20	Radix Cynanchi	1		bitter、salty、cold	stomach、liver、kidney

### 3.2 Systematic review

#### 3.2.1. Inhibition of excitatory amino acid toxicity

Glutamate (Glu) is the primary excitatory neurotransmitter in the adult mesolimbic system. They play an essential role in nerve conduction, neuronal excitability, and transmitter release. Following cerebral ischemia, stimulation with NMDA leads to calcium overload and the release of large amounts of glutamate, which may lead to excitotoxicity, thereby promoting mitochondrial metabolic damage, oxidative stress, free radical production, and ultimately neuronal death.

#### 3.2.2. Reducing inflammation

The role of inflammation in the pathology of ischemic stroke. Loss of brain energy, neuronal necrosis and apoptosis all lead to the mobilization of inflammatory cells and the release of large amounts of inflammatory factors, such as interferon (IFN)-γ, NF-κB, IL-1 and TNF-α. These inflammatory factors activate the immune response and promote the release of additional inflammatory factors, which in turn exacerbate the inflammatory reaction leading to encephalopathy.

#### 3.2.3. Anti-oxidative stress

Oxidative stress is defined as tissue injury caused by excessive ROS accumulation or decreased ROS scavenging during cerebral ischemia. Reperfusion leads to the production of massive amounts of radicals, which can attack the phospholipid components of brain tissue and accelerate brain cell necrosis leading to brain death.

#### 3.2.4. Protects against apoptosis of nerve cells

Apoptosis is a cell death process that maintains the stability of the internal environment and is actively sought after by genetically controlled cells to better adapt to their survival environments. Ischemic in the brain leads to the massive apoptosis of neuronal cells. The inhibition of apoptosis during cerebral ischemia may be ideal for rescuing neurons in the penumbra and ensuring their survival, as well as being an important measure in the treatment of cerebral ischaemic.

#### 3.2.5. Other

In addition to the above mechanisms, heat-clearing herbs have also been shown to exert cerebral protective effects against ischemic brain injury through other pathways, such as the activation of the PGC-1α/Nrf2 signalling pathway by red peony to promote vascular renewal; Sun [7] et al. found that paeoniflorin inhibited the reduction of Na^+^-K^+^-ATPase activity after reperfusion, increased NO levels and regulated active nitrogen content thus exerting anti-ischemic stroke effects; Lignans [8] can inhibit the proliferation and migration of vascular smooth muscle cells caused by angiotensin II. Table 2 summarizes the mechanisms of the anti-ischemic brain injury caused by the active ingredients of heat-clearing herbs.

**Table 2.** Mechanism of anti-ischemic brain injury by active ingredients of heat-clearing herb.

A: Inhibition of excitatory amino acid toxicity B: Reducing inflammation C: Anti-oxidative stress D: Protects against apoptosis of nerve cells E: Mitochondrial dysfunction F: Protecting the blood-brain barrier G: Other					
Activity classification	Active ingredients	Source	Impact	Mechanism of action	Documentary sources
Terpenoids	Total glycoside of radi	Radix Paeoniae Rubra	A	Increase the activity of Na^+^-K^+^-ATPase and Ca^2+^-ATPase in brain tissue, improve the impaired energy metabolism in brain tissue, and inhibit intracellular calcium overload	[9]
			C	Reduces MDA content in brain tissue and inhibits LDH activity	
	Paeoniflorin		B	Reduction of the presence of ED1, IL-1β, TNF-α, ICAM-1 in apoptotic cells	[10]
			B	Inhibiting NF-κB expression, reducing glial cell activation due to chronic cerebral hypoperfusion, and preventing hippocampal neuronal loss	[11]
			D	Reversal of the Glu-induced decrease in the Bcl-2/Bax ratio	[12]
			A	Blocking Glu-induced intracellular calcium spillover and inhibiting CaMKII expression	
			B	Inhibits astrocyte and microglia hyperactivation and blocks upregulation of TNF-a, IL-1β, iNOS, COX2, and 5-LOX	[13]
	Geniposide	Gardeniae Fructus	F	Reduced water channel protein-4 levels and significantly inhibited MMP-9 and MMP-2 expression	[14]
			DF	Regulation of VEGF and ANG expression, with VEGF reducing BBB permeability and ANG inhibiting endothelial cell apoptosis	[15]
			D	Reduced expression of HIF-1α and related gene RTP801 mRNA in the CA1 region of the hippocampus	[16]
			D	Up-regulation of Bcl-2 mRNA and inhibition of the expression of apoptosis-related proteins P53, Caspase-3, and Caspase-9	[17]
			B	Interferes with P2Y14 receptor expression and inhibits the downstream ERK1/2 signaling pathway and the release of IL-8, MCP-1, and IL-1β	[18]
			D	Reduction of HIF-1α expression in the hippocampal CAI region	[19]
			B	Inhibits hypoxic/reoxygenated SHY-5Y cell protein myeloid differentiation factor 88, NFκB, ERK1/2, IκB activation and inhibits TLR4 pathway to exert anti-inflammatory effects	
			A	Down-regulation of GRIN2C gene expression at the glutamate receptor inhibits the interaction of excitatory amino acids with excitatory amino acid receptors	[20]
			B	Down-regulates the expression of the purinergic receptor P2Y14, which is involved in vascular endothelial cell injury repair, activates the rat sarcoma (Ras) protein, further activates the MAPK signaling pathway, regulates the phosphorylation of ERK1/2 and reduces the expression of the proteins Raf-1, MEK1/2, and ERK1/2 in a hypoxic glucose deprived cell model	[18]
			B	Reduced expression of ICAM-1, VCAM-1, and E-selectin in brain tissue and reduced adhesion between leukocytes and vascular endothelial cells	[21]
	Crocin	Gardeniae Fructus	C	Reduces MDA content and increases SOD and GPx activity in the ischemic cortex	[22]
			F	Inhibition of GRK2 translocation from the cytoplasm to the cell membrane and expression of cortical p-ERK1/2, cortical GFAP and MMP-9	[23]
			E	Increased mitochondrial membrane potential, decreased intracellular calcium ion concentration, inhibited mitochondrial dynamin-related protein 1 (Drp1) expression, and upregulated optic dystrophy protein 1 (Opa1) expression in SH-SY5Y cells induced by OGD injury	[24]
	Crocetin	Gardeniae Fructus	C	Increase SOD activity and decrease NO and MDA levels in brain tissue	[25]
	Harpagide	Figwort Root	D	Down-regulation of C/EBP homolog, cysteine aspartate-12, glucose regulatory protein 78 gene expression	[26]
	Betulinic acid	Cortex Moutan	C	Inhibits ROS and reduces NO levels and eNOS activity	[27]
	Oleanolic acid	Cortex Moutan、Figwort Root	C	Activation of Nrf2 reduces the expression of nNOS and iNOS during brain hypoxia and protects neurons from microglia-induced oxidative damage	[28]
	Paeoniflorin	Cortex Moutan、Radix Paeoniae Rubra	A	Inhibits the release of Glu, aspartic acid	[10]
Alkaloids	Coptisine	Coptidis Rhizoma	D	Reducing the number of apoptotic neurons and increasing the expression of bcl-2 protein	[29]
	Berberine	Coptidis Rhizoma	D	Enhanced p55γ promoter activity, increased Akt phosphorylation, inactivated Bad, and reduced the expression of pro-apoptotic caspase-3	[30]
			D	Reduced the expression levels of autophagy-related proteins SIRT1, BNIP3 and Beclin-1, and also reduced the expression levels of p-AMPK and p-mTORC2 (Ser2481) in H9c2 cells after hypoxia-reperfusion	[31]
			C	Activation of PPARδ and promotion of Nrf1, Nrf2, and quinone oxidoreductase 1 expression	[32]
			G	Up-regulation of brain-derived neurotrophic factor BDNF mRNA expression in the hippocampus of rats with cerebral ischemia-reperfusion injury	[33]
			D	Down-regulation of CNPY2-regulated endoplasmic reticulum stress reduces caspase-3 levels in the ischemic semidark zone	[34]
			D	Decreased Bax and caspase-3, down-regulated endoplasmic reticulum stress-related proteins GRP78 and CHOP, and down-regulated autophagy-related protein LC3-II/LC3-I ratio	[35]
			B	Reduces high mobility group protein 1, Toll4 and NF-κB levels and decreases TNF-α, IL-1β and IL-6 levels	[36]
			D	Antagonizes the degradation of RB1 mRNA, thereby stabilizing Rb protein levels, blocking the cell cycle, and promoting neuronal cell survival in ischemic brain injury	[37]
			B	Reduced AQP4 expression in brain tissue	[38]
Flavonoids	Baicalin	Scutellariae Radix	C	Enhance the activity of SOD, GSH, and GSH-PX and reduce the level of MDA	[39]
			C	Inhibition of exogenous and endogenous peroxynitrite anion-induced neurotoxicity protects SH-SY5Y neural cell line	[40]
			EF	Reduced expression of MMP-9 protein and mRNA and up-regulated expression of the tight junction protein including	[41]
			B	Inhibition of the TLR4/MYD88/NF-κB-signalling pathway reduces the release of inflammatory factors from HBMEC induced by OGD injury	[42]
			B	Reverses the polarization state of microglia, thereby reducing the inflammatory response and damage caused by microglia xenoreactivation after ischaemic brain injury	[43]
			D	Promotes the expression of brain-derived BDNF and inhibits the expression of Caspase-3 protein and mRNA	[39]
	Quercetin	Gardeniae Fructus、Lonicerae Japonicae Flos、Cortex Moutan	C	Activation of the Nrf2 signalling pathway attenuates ischemia-reperfusion-induced neuronal cell injury	[44]
			F	Elevated expression levels of ZO-1, Occludin and Claudin-5	[45]
	Luteolin	Lonicerae Japonicae Flos、Cortex Moutan、Scutellariae Radix	B	Interference with the JNK signalling pathway and activation of AP-1 in microglia to reduce the LPS-induced increase in IL-6 levels	[46]
			C	Inhibition of the HIF-1α/NLRP3 signalling pathway inhibits neuronal apoptosis and significantly increases SOD activity and reduces MDA content	[47]
	Chrysin	Scutellariae Radix	C	Expression of Nrf2 and HO-1 was upregulated	[48]
	Kaempferol	Lonicerae Japonicae Flos、Cortex Moutan	B	Downregulation of TLR4, NF-κB, p38, JNK, and Akt, and reduction of LPS-induced inflammatory mediators NO, PGE2, TNF-α, IL-1β, and ROS	[49]
	Apigenin	Scutellariae Radix、 Radix Paeoniae Rubra、 Cortex Moutan	G	Modulates the content of adhesion factors in reactive astrocytes and effectively reduces TNF-α-induced VCAM-1 mRNA and protein expression	[50]
Phenylpropanoid	Resveratrol	Cortex Moutan、Cassiae Semen	D	Reduced caspase-3 and GFAP expression levels, neuronal damage, and neurodegeneration in the hippocampus and cortex	[51]
			C	Decrease NMDA and iNOS levels, increase SOD levels and decrease MPO levels	[52]
	Chlorogenic	Gardeniae Fructus、 Coptidis Rhizoma、 Lonicerae Japonicae Flos	B	Decrease ICAM-1 and VCAM-1 level in brain tissue, reduce the release of inflammatory mediators and cytokines, increase EPO and HIF-1a levels in brain tissue; increase NGF expression in brain tissue and activate the self-protection mechanism of brain cells	[53]
			D	Up-regulation of Bcl-2 gene expression and down-regulation of caspase-3 gene expression	[54]
	Caffeic acid	Scutellariae Radix、 Lonicerae Japonicae Flos、 Cortex Moutan	BC	SOD activity was significantly increased, MDA content and NF-κBp65-positive cell expression were significantly decreased	[55]
			B	Reduced 5-LO expression reduces leukotriene production after an ischemic stroke	[55]
	Sodium ferulate	Scutellariae Radix、 Lonicerae Japonicae Flos、Coptidis Rhizoma	B	Reduced TNF-a, IL-1β, and IL-6 levels in serum and brain tissue and inhibited NF-κBp65 translocation from cytoplasm to nucleus	[56]
	Verbascoside	Scutellariae Radix	D	Inhibition of mitochondrial apoptotic protein caspas3 and PARP shearing, downregulation of Bax, and increase in Bcl-2 protein expression	[57]
Anthraquinone	Emodin	Cassiae Semen	B	Inhibition of NF-κB signalling pathway and reduction of inflammatory factor secretion	[58]
	Rhein		B	Activation of PI3K/AKT/mTOR pathway and reduction of TNF-α, IL-6, and IL-1β levels in cell culture supernatants	[59]
	Chrysophanol		C	Reduced serum MDA levels and up-regulated serum SOD, GSH-Px, and CAT levels in mice to improve antioxidant capacity	[60]
Essential oil	Paeonol	Cortex Moutan、 Radix Paeoniae Rubra	B	Significant reduction in macrophage-microglia cytoplasmic antigen (ED1) and IL-1β-positive cells in the infarct region with inhibition of microglia	[61]
			A	Acts on the NMDA receptor to inhibit the massive Ca^2+^ influx by reducing the binding of Ca^2+^ to the NMDA receptor to maintain the stability of intracellular Ca^2+^ content	[62]
			D	Decreased levels of apoptosis-inducing factor (AIF) in the cytoplasm	[63]
			D	Reduction of TLR4 expression significantly ameliorates cell damage in the rat cortical area	[64]
			C	Lower plasma ET levels, lower brain tissue iNOS levels, and NO levels	[65]
	Eugenol	Cortex Moutan、 Figwort Root	BC	GSH, SOD, and CAT activities were significantly increased, MDA content was significantly decreased and NF-κB expression was down-regulated	[66]
	Vanillin	Figwort Root、 Scutellariae Radix	B	Regulation of SIRT1/HMGB1/TLR4/MyD88/NF-κB signalling pathway inhibits HIBI-induced neuroinflammation	[67]
Polysaccharides	Polysaccharides	Figwort Root	BC	Increased SOD activity, reduced MDA, NO, NOS, IL-1β and TNF-α levels; improved reduction in ERK protein expression and increased JNK and p38 protein expression	[68]
			CD	Reduced NO and MDA concentrations and LDH leakage release levels; up-regulated SOD, GSH-Px, and CAT enzyme activities to improve antioxidant capacity; up-regulated Bcl-2 expression and down-regulated Bax expression	[69]
			D	Up-regulation of SERCA2 maintains calcium homoeostasis and inhibits endoplasmic reticulum stress-mediated apoptosis	[70]
			CD	Increase cell survival and SOD activity; decrease LDH level and apoptosis rate; decrease CHOP, Caspase-12, and GRP78 protein expression levels	[71]
			G	Improves cortical CBF and blood flow after 2h of ischemia	[72]
		Radix Paeoniae Rubra	G	Induces high VEGF/VEGFR-2 expression through activation of the PGC-1α/Nrf2 signalling pathway and promotes vascular neogenesis	[73]
		Cassiae Semen	B	Reduced TNF-α and IL-6 levels, mtDNA levels, and p-STING/STING and cGAS protein levels in the cytoplasm of the cortical area inhibited mtDNA-mediated activation of the STING pathway	[74]

### 3.3 Screening results of active ingredients of heat-clearing Chinese medicines

All the chemically active ingredients retrieved from the TCMSP platform were selected for screening conditions of OB ≥ 30% and DL ≥ 0.18. The results yielded 29 related components of Radix Paeoniae Rubra, 2 components Rehmanniae Radix Praeparata, 9 Figwort Root, 11 Cortex Moutan, 36 Scutellariae Radix, 14 Coptidis Rhizoma, 15 Gardeniae Fructus, 14 Cassiae Semen, and 23 of Lonicerae Japonicae Flos, with which the relevant targets were 99 of Radix Paeoniae Rubra, 30 of Rehmanniae Radix Praeparata, 53 Figwort Root, 167 Cortex Moutan, 117 Scutellariae Radix, 181 Coptidis Rhizoma, 196 Gardeniae Fructus, 65 Cassiae Semen, and 212 Lonicerae Japonicae Flos. The structural formulas of the active ingredients of each drug are presented in Supplementary Appendix 1.

### 3.4. Construction of drug component-target-pathway interaction network diagrams

**Figure 3.**
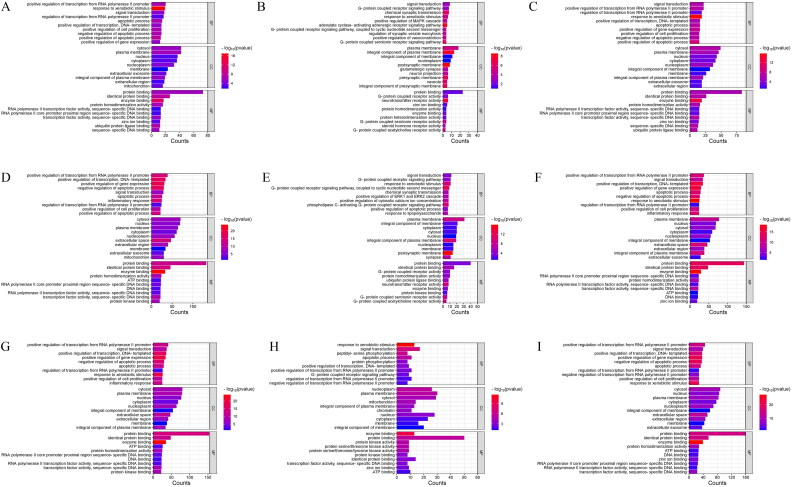
GO enrichment analysis results. A: Radix Paeoniae Rubra B: Rehmanniae Radix Praeparata C: Figwort Root D: Cortex Moutan E: Scutellariae Radix F: Coptidis Rhizoma G: Gardeniae Fructus H: Cassiae Semen I: Lonicerae Japonicae Flos

All base targets of each heat-clearing herbal medicine for ischemic encephalopathy were included, and GO functional enrichment analysis was performed. Based on the GO enrichment analysis of biological processes (including biological process (BP), cellular composition (CC) and molecular function (MF)), the top 10 with the smallest *P*-value in each part were screened as prominent biological processes (Figure 3), and the GO enrichment result analysis is shown in Supplementary Appendix 2.

**Figure 4.**
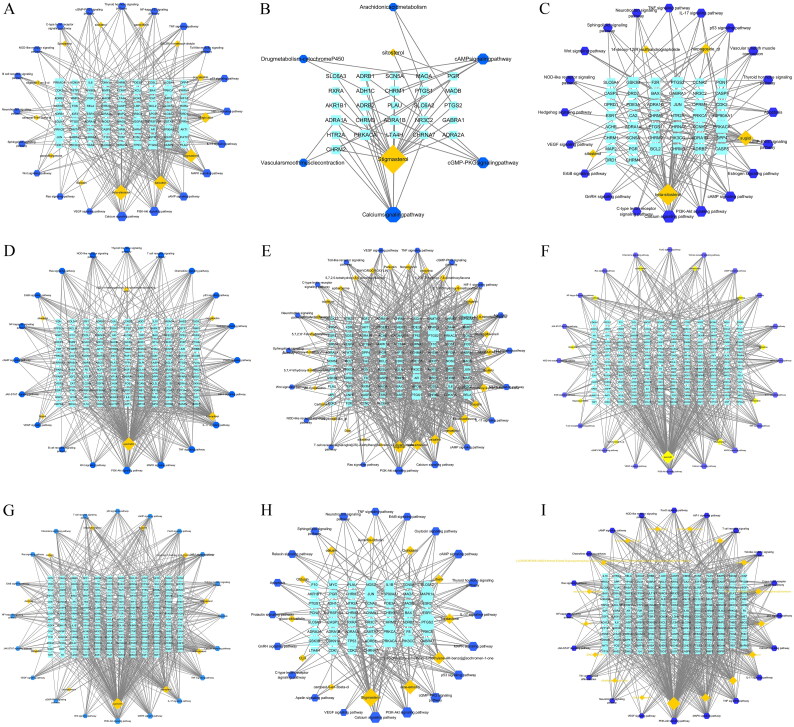
Drug component-target-pathway interaction network diagrams. A: Radix Paeoniae Rubra B: Rehmanniae Radix Praeparata C: Figwort Root D: Cortex Moutan E: Scutellariae Radix F: Coptidis Rhizoma G: Gardeniae Fructus H: Cassiae Semen I: Lonicerae Japonicae Flos.

We used Cytoscape 3.7.2 software to construct the network diagrams of drug component-target-pathways between each drug pair and ischemic encephalopathy, and the results are shown in Figure 4. The shapes and sizes of the components represented in the diagrams were adjusted according to their degree values, and it was found that components with larger degree values played a more important role in the network diagrams. The results are presented in Supplementary Appendix 2.

A: Radix Paeoniae Rubra B: Rehmanniae Radix Praeparata C: Figwort Root D: Cortex Moutan E: Scutellariae Radix F: Coptidis Rhizoma G: Gardeniae Fructus H: Cassiae Semen I: Lonicerae Japonicae Flos

According to the analysis of the median value of the network diagram it was concluded that the main components of the anti-ischemic encephalopathy of the purgative herbs were beta-sitosterol, quercetin, and Stigmasterol, which acted mainly on the key targets such as RELA, AKT1, JUN, PRKACA, PTGS2, RAF1, and CHUK; Its active ingredients are mainly involved in signaling pathways such as Calcium, PI3K-Ak, MAPK, cAMP, IL-17, HIF-1, TNF, T cell receptor, NF-kappa B, JAK-STAT; Based on the results of GO analysis, it was concluded that purgative herbs may exert their anti-ischemic encephalopathic effects through a variety of pathways such as apoptotic, positive regulation of cell proliferation, inflammatory response, G-protein coupled receptor, positive regulation of cytosolic calcium ion concentration, positive regulation of ERK1 and ERK2 cascade and chemical synaptic transmission.

## 4. Discussion

Ischaemic cerebrovascular disease seriously damages human health and is characterized by high morbidity and mortality rates; therefore, there is an urgent need to find ways to reduce cerebral morbidity and injury rates. Based on the aetiology and pathogenesis of ischaemic cerebrovascular disease, and under the guidance of the principle of diagnosis and treatment in Chinese medicine, the disease can be approached from the perspectives of clearing the channels and removing phlegm, benefiting qi and activating blood circulation, strengthening the spleen and tonifying the kidneys, dredging the liver and resolving depression, and clearing heat and removing toxins; Summarising the clinical lesion characteristics of stroke disease, treatment success or failure experience, and combining the new viewpoints of modern medical research on the ischemic damage process of stroke disease, and the in-depth understanding of the pathogenesis of stroke disease, Academician Wang Yongyan put forward the theory of the ‘toxic damage to the brain and collateral’ hypothesis, which believes that draining toxins and removing heat, nourishing the blood and activating collaterals, and regulating and regulating camping and guarding the hair method can make the brain and the spirit to be nourished, and the spirit and the machine can be restored. In this review, we summarized the signalling pathways involved in ischaemic encephalopathy and briefly classified them according to their specific pathophysiological roles in excitotoxicity, mitochondrial dysfunction, oxidative stress, neuroinflammation and apoptosis. As these signalling pathways are interconnected, combinatorial therapeutic targets for anti-ischaemic encephalopathy can identified. In this study, we used informative quantitative statistical methods to screen commonly used heat-clearing herbs against ischemic encephalopathy and applied network pharmacology to describe the relationships between active substances, compound targets and signalling pathways.

The results of Part One study show that TCM plays an irreplaceable role in the management of ischemic encephalopathy, effectively improving the patient’s condition and facilitating disease healing. In terms of drug classes, heat-clearing herbs have proven efficacy in the prevention and treatment of ischemic strokes. Most of the herbs used for clearing heat are cold, bitter and sweet in nature, and the cold ones activate blood stasis, clearing turbidity and dispelling phlegm; the bitter taste is good at removing turbidity and clearing the internal organs, while the sweet taste is moist and good at nourishing dryness; enter the liver, heart, and spleen meridians to nourish the liver, calm the liver, tonify the qi, invigorate the blood, and invigorate the spleen to resolve phlegm and lower turbidity. Knowledge of the medication rules for the treatment of ischemic stroke can effectively guide clinicians regarding the therapeutic use of medication in patients with ischemic encephalopathy.

In this study, a total of 9 heat-clearing traditional Chinese medicines were screened, and the components, targets and pathways with the highest degree values were screened through the network diagram analysis of ‘drug components – intersecting targets – signalling pathways,’ and the main components for the treatment of ischemic encephalopathy were screened as β-sitosterol, stigmasterol and quercetin. β-Sitosterol has been in the spotlight in recent years for its ability to indirectly protect tissues and organs from ischaemia/reperfusion injury by generating anti-oxidant effects through pathways such as promoting the glutathione redox cycle, for example, its angiogenic effects, which can effectively restore motor function in gerbils with ischaemia/reperfusion injury; Some findings have shown that soy sterols have anti-inflammatory and antioxidant properties; Quercetin is a naturally occurring flavonoid that has anti-inflammatory, anti-apoptotic, antioxidant, anti-infectious and anti-tumour effects, promotes vasodilation and is used in the treatment of metabolic and cardiovascular diseases. RELA, AKT1, JUN, PRKACA, PTGS2, RAF1 and CHUK were identified as four key targets associated with ischemic encephalopathy. GO enrichment analysis showed that the anti-ischemic encephalopathic effects of purgatives were associated with major biological processes such as apoptosis, cell proliferation, inflammatory response, chemical synaptic transmission and calcium ion concentration regulation; KEGG screened out therapeutic processes mainly involving Calcium, PI3K-Ak, MAPK, cAMP, IL-17, HIF-1, TNF, T cell receptor, NF-kappa B, JAK-STAT signalling pathways; Ischemia-induced excitotoxicity has been central to the study of stroke, where excessive release of the excitatory neurotransmitter glutamate after ischaemia leads to cell death. This is associated with an increase in intracellular calcium ion concentration. A significant increase in glutamate concentration thereby over-activating the NMDA receptor opens the calcium channel regulated by this receptor, causing massive calcium inward flow and excitotoxic effects on ischemic neurons; Within a few hours after the onset of cerebral ischaemia, the ischaemic semi-dark zone region is mainly dominated by neuronal apoptosis. This directly affects the area of cerebral infarction as well as the recovery of neurological function in the brain. PI3K-Akt signalling pathway and MAPK signalling pathway, as an important signalling pathway of cell proliferation *in vivo*, are involved in the regulation of a wide range of biological processes such as cell proliferation, survival, and apoptosis, and play an important role in cerebral ischaemic injury; cAMP is associated with transmitters released in synaptic connections between sensory and motor neurons. The cAMP response element binding protein (CREB) is a target of PKA and is thought to play a crucial role in signal transduction that promotes neuronal survival and differentiation. CREB phosphorylation is induced primarily by cAMP-dependent protein kinases. Numerous cellular and animal experiments have demonstrated that the cAMP/PKA/CREB signalling pathway regulates the inflammatory response and plays a neuroprotective role in ischemic brain injury. Inflammatory cytokines are directly involved as independent risk factors in the development of cerebral infarction, and the TNF and IL-17 signalling pathways are important signalling pathways that mediate the inflammatory response of the body. The JAK/STAT3 signalling pathway, a major inflammatory signalling pathway in the body, plays a key regulatory role in the development of ICS and is activated during ICS pathology. It has been shown that miR-216a can target down-regulate JAK gene, significantly inhibit STAT3 activation, reduce the overexpression of inflammatory enzyme-inducible nitric oxide synthase, matrix metalloproteinase 9, and inflammatory cytokines tumour necrosis factor α and IL-1β due to ICS, counteract neuroinflammation, reduce cerebral infarctions, and facilitate neuronal cell survival to ameliorate neurological deficits. Microglia are immune-responsive cells of the central nervous system that play key roles in neuroinflammatory processes. NF-κB is a central regulator of the neuroinflammatory response and is responsible for microglia M1 and M2 polarization. In the early stage of cerebral ischemia, TNF-α, IL-6, and IL-1β are activated as inflammatory factors downstream of the NF-κB signalling pathway, and high concentrations of TNF-α, IL-6, and IL-1β in turn activate the expression of NF-κB, which promotes the polarization of microglia towards the M1 phenotype and the expression of related chemokines. Some studies have shown that the activity of NF-κB is related to the severity of stroke, and down-regulation of NF-κB expression can attenuate cerebral oedema and neurological damage, reduce apoptosis, and decrease infarct size. HIF-1α is a transcription factor that plays an important role in the regulation of various physiological and pathological conditions such as inflammation, energy metabolism, and apoptosis. HIF-1α, whose expression is rapidly induced after hypoxia/ischaemia, plays an important role in a variety of diseases by regulating important processes such as erythropoiesis, angiogenesis, glycolysis and catecholamine metabolism. HIF-1α also polymerizes with HIF-1β to form HIF-1, which mediates the transcriptional activation of VEGF, promotes the proliferation of vascular endothelial cells, enhances vascular permeability, and facilitates angiogenesis in damaged brain tissue.

## 5. Conclusion

In conclusion, based on the active ingredients of heat-clearing traditional Chinese medicines and their therapeutic status studied at home and abroad, heat-clearing traditional Chinese medicines have achieved certain results and have great potential for the protection and prevention of ischemic encephalopathy. The results of the network pharmacological studies are similar to the anti-ischemic encephalopathy mechanisms of the active ingredients of the purgative herbs that we have listed, some of which protect cerebrovascular tissues directly by improving vascular permeability and reducing the area of infarcted tissues, whereas others produce protective effects through molecular signalling pathways (Calcium, PI3K-Ak, MAPK, cAMP, IL-17, HIF-1, TNF, T cell receptor, NF-kappa B, and JAK-STAT). It can be seen that the components of heat-clearing Chinese medicines can exert cerebroprotective effects through multiple pathways. This provides us with a reference for further development and study of heat-clearing Chinese medicines in the treatment of ischemic cerebrovascular diseases. The credibility of the current clinical studies of Chinese medicine is mixed, and there is a lack of reliable evidence-based medical evidence. Strengthening the applied research of Chinese medicine in the field of modern medicine and conducting clinical trials with greater credibility and validity are important directions for the future development of Chinese medicine for the prevention and treatment of ischemic cerebrovascular disease.

## Supplementary Material

Supplemental Material

## Data Availability

These data were derived from the following resources available in the public doma: https://db.yaozh.com/chufang; http://www.zhongyoo.com/fangji/; https://www.cnki.net/; https://pubmed.ncbi.nlm.nih.gov/?term=; https://old.tcmsp-e.com/tcmsp.php.
