# Supplementary material for: Substance basis and pharmacological mechanism of heat-clearing herbs in the treatment of ischaemic encephalopathy: a systematic review and network pharmacology
Source: Ann Med. 2024 Jan 29;56(1):2308077. doi: 10.1080/07853890.2024.2308077 (PMC10826791; doi:10.1080/07853890.2024.2308077)
Supplement: Supplemental Material [file IANN_A_2308077_SM3746.zip › Supplementary Appendix 1.docx]

Chemical structure formulae of active ingredients of heat-clearing Chinese medicines

A: *Paeonia lactiflora* Pall.

B: *Rehmannia glutinosa* (Gaertn.) DC.

C: *Scrophularia ningpoensis* Hemsl.

D: *Paeonia ×suffruticosa* Andrews

E: *Scutellaria baicalensis* Georgi

F: *Coptis chinensis* Franch.

G: *Gardenia jasminoides* J.Ellis

H: *Senna tora*(L.) Roxb.

I: *Lonicera praeflorens var. japonica* H.Hara

**A**

**B**

**C**

**D**

**E**

**F**

**G**

**H**

**I**
